# Modeling long-distance airborne transmission of highly pathogenic avian influenza carried by dust particles

**DOI:** 10.1038/s41598-023-42897-2

**Published:** 2023-09-27

**Authors:** X. D. Nguyen, Y. Zhao, J. Lin, J. L. Purswell, T. Tabler, B. Voy, S. Hawkins, J. D. Evans

**Affiliations:** 1 Animal Science, The University of Tennessee, Knoxville, USA; 2 Poultry Research Unit, USDA Agricultural Research Service, Mississippi State, MS USA; 3 Biosystems Engineering and Soil Sciences, The University of Tennessee, Knoxville, USA

**Keywords:** Microbiology, Environmental sciences, Engineering

## Abstract

Highly pathogenic avian influenza (HPAI) is continuously causing significant economic losses with massive poultry depopulations. Airborne transmission of HPAI was suspected, as initial bird mortalities were reported near air inlets of poultry houses. In addition, infected farms were distant, indicating that the viruses carried by dust particles might help the viruses travel for long distances in the environment. The objective of this study focused on simulating the airborne transmission of HPAI by using computational modeling to assess the risk of airborne and deposited avian influenza (AI) carried by poultry-litter dust particles. The Hybrid Single-Particle Lagrangian Integrated Trajectory (HYSPLIT) modeling was used in this study. Data from 168 infected cases in the Mid-Western area of U.S. were obtained from the Animal and Plant Health Inspection Service (APHIS) and Watt Poultry. The concentration simulation modeling was performed to estimate the airborne and deposited AI concentration carried by PM_2.5_ dust particles. Results showed that concentrations of airborne AI, deposited AI, and combined AI transmitted to other farms in a day were lower than the minimal infective dose for poultry. In most of the scenarios, the predicted probability of infection showed that Iowa-infected farms and turkey poultry houses had the highest infection probability. The findings may provide an understanding of the risk of airborne HPAI virus carried by dust particles and suggest the factors that influence long-distance airborne transmission.

## Introduction

The U.S. poultry industry is among the world's largest poultry producers. It includes meat products from turkeys, broilers, and eggs from laying hens. The combined values of these products exceeded 35 billion U.S. dollars in 2020^1^. Poultry products are affordable and important sources of daily protein. In addition, the U.S. poultry industry provides $555.9 billion in economic activity and about 2 million jobs for national populations^2^. However, this important industry is extremely vulnerable to infectious diseases caused by pathogenic microorganisms such as highly pathogenic avian influenza (HPAI). An example is the 2014–2015 HPAI outbreak in the Mid-Western U.S., resulting in a significant loss of over 50 million birds and 3.3 billion U.S. dollars^3^.

Avian influenza (AI) or bird flu refers to the infectious disease caused by the infection of influenza virus type A from *orthomyxoviridea*. These viruses are found in wild aquatic birds worldwide and, also can infect domestic poultry, and other bird and certain mammal species. Although avian influenza A viruses can infect wild aquatic birds' intestines and respiratory tracts, other species, such as wild ducks, may not become ill. Avian influenza A viruses are highly infectious among commercial poultry, and some strains can sicken and even kill certain domesticated bird species such as chickens, domestic ducks, and turkeys. As of November 2022, approximately 50 million birds including 265 commercial flocks and 358 backyard flocks have been affected by the 2022 AI outbreak^4^. AI viruses can be found in infected birds' saliva, nasal secretions, and feces. Transmission of the pathogen to naive (susceptible) birds can result from direct contact between birds or by indirect contact with virus-contaminated fomites^5^.

Due to the severity of the disease, AI viruses are divided into two groups including low pathogenic avian influenza (LPAI) and high pathogenic avian influenza (HPAI). Low pathogenic avian influenza viruses produce little or moderate illness in laying hens and broilers (such as ruffled feathers and a drop in egg production). The majority of avian influenza viruses are low pathogenic, causing little symptoms of illness in infected wild birds. Some low-pathogenic viruses in chickens can evolve into highly pathogenic avian influenza viruses. On the other hand, infected chickens suffer from severe sickness and a high death rate due to HPAI viruses. Only a few avian influenza A(H5) and A(H7) viruses are HPAI type A viruses, while the vast majority of avian influenza A(H5) and A(H7) viruses circulating in birds are LPAI viruses. In hens, HPAI type A(H5) or A(H7) virus infections can produce sickness that affects several internal organs, with mortality rates ranging from 90 to 100%, frequently within 48 h^6^. Infections of HPAI A(H5) and A(H7) viruses in poultry can transmit to wild birds, resulting in the additional geographic spread of the virus when the birds migrate. HPAI viruses are transmitted mainly through direct contact infection. However, with the initial bird mortalities reported near air inlets of poultry houses, there is a high chance that HPAI viruses were transmitted into poultry houses by airborne pathway.

In the poultry house, the major components of the air include gases, odors, and dust and droplet nuclei carrying microorganisms. AI viruses are first secreted via birds’ nasal secretions, feces, and saliva. The bird secretions can either be dried and suspended in the air for a long period of time or deposited on the poultry litter surface. The deposited secretions which carry AI are then mixed with poultry litter particles and re-aerosolized into the air by bird activities. Both droplet nuclei and dust particles that carry AI may then be distributed into the poultry house environment and transmitted from barn to barn via ventilation system and transport of air.

At susceptible farms, the AI can be sucked in through the ventilation system and be distributed inside the farms. The airborne HPAI viruses are then deposited onto the surface of poultry litter on which they can survive up to 5 days at 24 °C^7^. In previous studies, authors have reported that most airborne AI viruses are found in dust particles as small as 1–5 µm in size^8, 9^ at 0.5 m away from poultry housing. It is important to note that fine dust particles (or dust particles with diameters that are generally 2.5 µm and smaller) can travel hundreds of miles^10^. With the long dispersion range, the AI viruses carried by fine dust particles can be a possible transmission pathway of HPAI.

To determine the possibility of long-distance airborne transmission of HPAI carried by poultry dust particles, this study aims at simulating the airborne transmission of HPAI by using the Hybrid Single-Particle Lagrangian Integrated Trajectory model (HYSPLIT) to assess the risk of airborne and deposited AI carried by poultry litter dust particles. Compared to other models such as Computational fluid dynamics (CFD) which is also able to simulate the flow of the air, the advantage of HYSPLIT is to integrate the meteorological data into the model which improves the accuracy of the simulation. In the study, 168 infected commercial poultry cases (72% of the national total of the infected commercial farms) in the Mid-Western U.S. were focused on, and data from the period of February 08th, 2022 to May 22nd, 2022, when cases appeared in the area were included in the model. Infected backyard birds accounted for a small number of infected birds with a low number of infected farms, and therefore, were not included in this study.

## Results

### Infected farm data

A total of 168 infected case data in the Mid-Western U.S. which accounted for about 72% of the national total of commercial farm infections were utilized in the study (Table S2). The time period of February 08th, 2022 to May 22th, 2022 were included in the model. Physical address, county and state, infection confirmation date, and the number of birds infected were collected.

### Minimal infective doses

The infective dosage, lung capacity, breathing rate, and exposure duration were all used to compute the MIDa and MIDd. Lung capacity and respiratory rate in turkeys are 7.7 × 10^–5^ m^3^ and 2.4 × 10^3^ times h^−1^, respectively, and 1.4 × 10^5^ m^3^ and 1.6 × 10^3^ times h^−1^ in laying hens^11^. Over a day of exposure, the resulting MIDa values were 210 EID_50_ m^−3^ for turkeys and 5880 EID_50_ m^−3^ for laying hens. MIDd values were 2837 EID_50_ m^−2^ for turkeys and 28,460 EID_50_ m^−2^ for laying hens.

### Viral concentrations in different types of poultry houses

**Figure 1:**
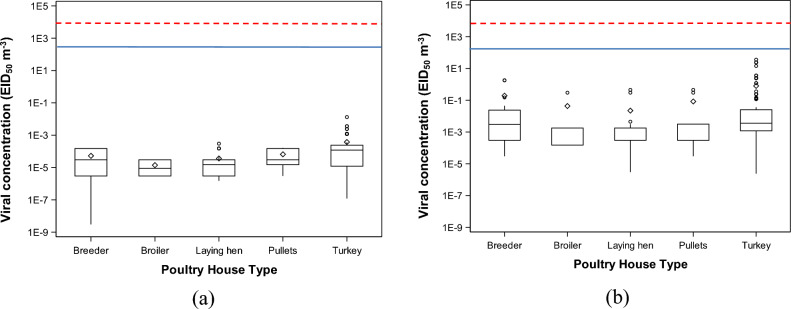
Concentrations of airborne highly pathogenic avian influenza (HPAI) viruses at the recipient farms. The concentration of avian influenza (AI) viruses is reported according to two categories (ceiling and default). The concentration of (**a**) airborne AI viruses in the default scenario, 50% egg infective dose (EID_50_) m^−3^; and (**b**) concentration of airborne AI viruses in the ceiling scenario, EID_50_ m^−3^. The blue solid line stands for the minimal infective dose of airborne AI (MIDa) values of turkey and the red dashed line stands for the MIDa values of laying hen.

**Figure 2:**
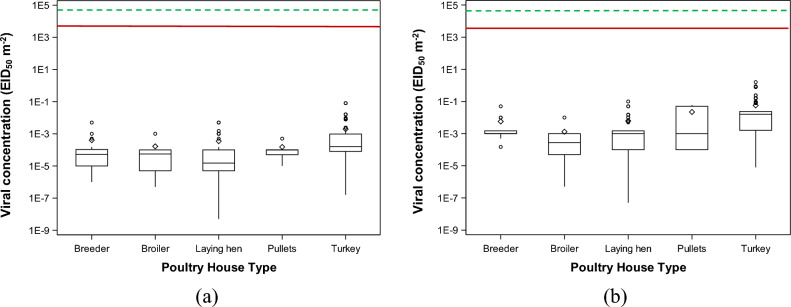
Concentrations of deposited highly pathogenic avian influenza (HPAI) viruses at the recipient farms. The concentration of avian influenza (AI) viruses is reported according to two categories (ceiling and default). The concentration of (**a**) airborne AI viruses in the ceiling scenario, 50% egg infective dose (EID_50_) m^−2^; and (**b**) deposited AI viruses in the default scenario, EID_50_ m^−2^. The red solid line stands for the minimal infective dose of deposited AI (MIDd) values of turkey and the green dashed line stands for the MIDd values of laying hen.

**Figure 3:**
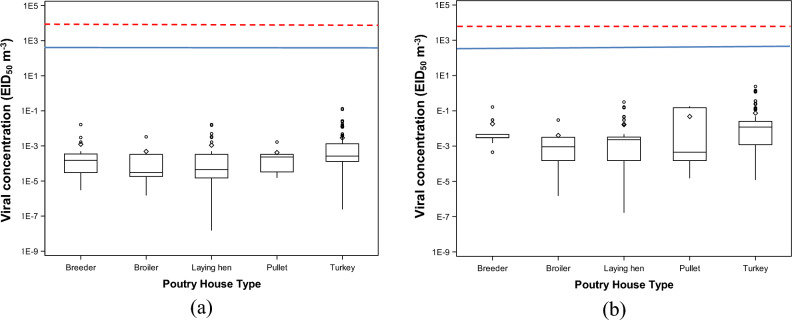
Combined concentrations of airborne and deposited highly pathogenic avian influenza (HPAI) viruses at the recipient farms. The concentration of avian influenza (AI) viruses is reported according to two categories (ceiling and default). The concentration of (**a**) AI viruses in the default scenario, 50% egg infective dose (EID_50_) m^−3^; and (**b**) concentration of AI viruses in the ceiling scenario, EID_50_ m^−3^. The blue solid line stands for the minimal infective dose of airborne AI (MIDa) values of turkey and the red dashed line stands for the MIDa values of laying hen.

The viral concentrations of AI in different types of poultry houses are reported in Fig. 1 (airborne AI), Fig. 2 (deposited AI), and Fig. 3 (combined concentrations of airborne and deposited AI). The figures show the viral concentrations carried by PM_2.5_ dust particles in 5 different types of poultry houses with 4 different scenarios including airborne ceiling, deposited ceiling, airborne default, and deposited default. Results show that in all categories, the viral concentrations were lower than MIDa and MIDd lines. This implies that under all scenarios, these commercial farms are likely not to have received viral loads above their MIDa and MIDd. This suggests that there is little risk of sludge development under both normal conditions and worst conditions.

### Farm infection probabilities in different states

**Figure 4:**
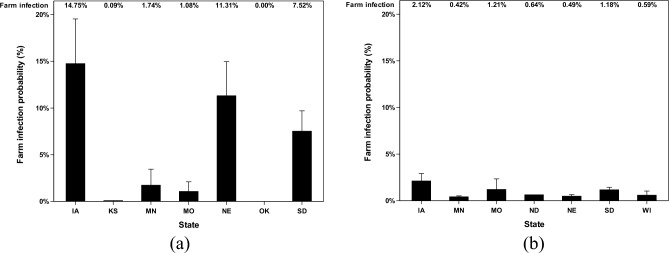
Farm infection probabilities of highly pathogenic avian influenza (HPAI) viruses at the recipient farms in default scenario. The farm infection probabilities of avian influenza (AI) viruses are reported according to two categories (deposited and airborne). Farm infection probabilities of (**a**) deposited AI viruses in default scenario, %; and farm infection probabilities of (**b**) airborne AI viruses in default scenario, %. The error bar stands for standard errors. X-axel abbreviations stand for states as follows IA (Iowa), KS (Kansas), MN (Minnesota), MO (Missouri), NE (Nebraska), SD (South Dakota), and WI (Wisconsin).

The farm infection probabilities of poultry farms that have received AI viruses from different locations in the Mid-Western area have been reported based on two categories, scenarios (default or ceiling) and transmission states (airborne or deposited). First, in the default scenario (Fig. 4), deposited AI data show three remarkable results that Iowa, Nebraska, and South Dakota farms have 14.8%, 11.3%, and 7.5% chance of being infected by AI viruses from the previously infected farm from the Mid-Western area. Minnesota, Missouri, Oklahoma, and Kansas farms have unremarkable percentages with the highest infection probability of 1.7% of having the chance to be infected from the Mid-Western area. Airborne AI data in the default scenario show low infection probability that Iowa farms, South Dakota farms, Missouri farms, and North Dakota farms have 2.1%, 1.2%, 1.2%, and 0.6% chance to be infected. Kansas, Minnesota, Nebraska, and Wisconsin have lower than 0.5% of chance that can be infected by AI.

**Figure 5:**
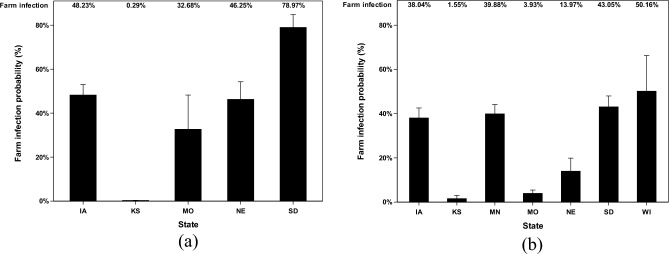
Farm infection probabilities of highly pathogenic avian influenza (HPAI) viruses at the recipient farms in ceiling scenario. The farm infection probabilities of avian influenza (AI) viruses are reported according to two categories (deposited and airborne). Farm infection probabilities of (**a**) deposited AI viruses in ceiling scenario, %; and farm infection probabilities of (**b**) airborne AI viruses in ceiling scenario, %. The error bar stands for standard errors. X-axel abbreviations stand for states as follows IA (Iowa), KS (Kansas), MN (Minnesota), MO (Missouri), NE (Nebraska), SD (South Dakota), and WI (Wisconsin).

The remaining results of ceiling scenarios (Fig. 5) of airborne and deposited AI data show significantly high infection probabilities with the highest probability of 79.0% in South Dakota. However, the chance of the ceiling scenario happening is relatively low compared to the default scenario. The remarkable infection probabilities of deposited AI viruses imply that in the ceiling scenario, susceptible farms in South Dakota can be infected by AI viruses from previously infected farms with a probability up to approximately 79.0%.

### Farm infection probabilities at different poultry house types

**Figure 6:**
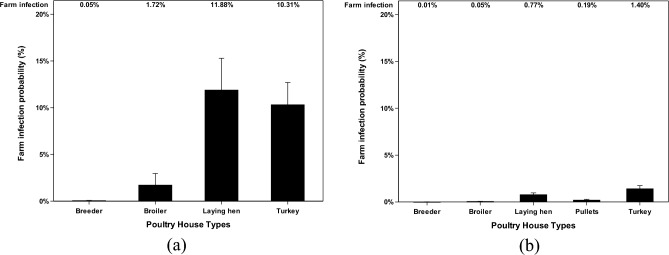
Farm infection probabilities of highly pathogenic avian influenza (HPAI) viruses at different poultry house types in default scenario. The farm infection probabilities of avian influenza (AI) viruses are reported according to two categories (deposited and airborne). Farm infection probabilities of (**a**) deposited AI viruses in default scenario, %; and farm infection probabilities of (**b**) airborne AI viruses in default scenario, %. The error bar stands for standard errors.

The farm infection probabilities of poultry farms in different poultry house types have been reported based on two categories, scenarios (default or ceiling) and transmission states (airborne or deposited). There were five poultry house types including breeder, broiler, laying hen, pullet, and turkey that were reported in this study. First, in the default scenario (Fig. 6), deposited AI data show two remarkable results that laying hen farms have 11.9% (the infection probability was measured each day) and turkey farms have 10.3% probability of being infected by AI viruses from the previously infected farm from the Mid-Western area. Broiler, breeder, and pullet farms have unremarkable percentages with the highest infection probability of 1.7% having the chance to be infected by AI from the Mid-Western area. Airborne AI data in the default scenario show low infection probability that turkey farms have 1.4%, laying hen farms have 0.8%, and pullet farms have 0.2% of being infected.

**Figure 7:**
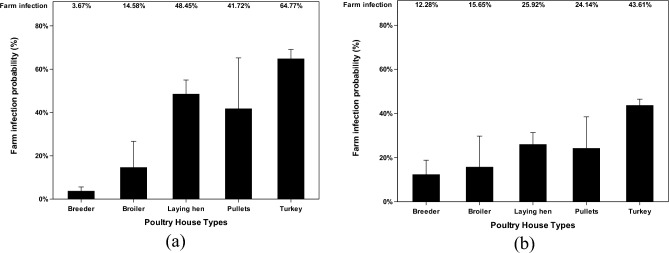
Farm infection probabilities of highly pathogenic avian influenza (HPAI) viruses at different poultry house types in ceiling scenario. The farm infection probabilities of avian influenza (AI) viruses are reported according to two categories (deposited and airborne). Farm infection probabilities of (**a**) deposited AI viruses in ceiling scenario, %; and farm infection probabilities of (**b**) airborne AI viruses in ceiling scenario, %. The error bar stands for standard errors.

The remaining results of ceiling scenarios (Fig. 7) of airborne and deposited data show significantly high infection probabilities with the highest probability of 64.8% in turkey farms. However, the chance of the ceiling scenario happening is relatively low compared to the default scenario. In all scenarios, turkey farms are the poultry house type that has the highest chance to get infected by AI with one exception in the deposited default scenario.

### Farm infection probabilities caused by combined AI concentration

**Figure 8:**
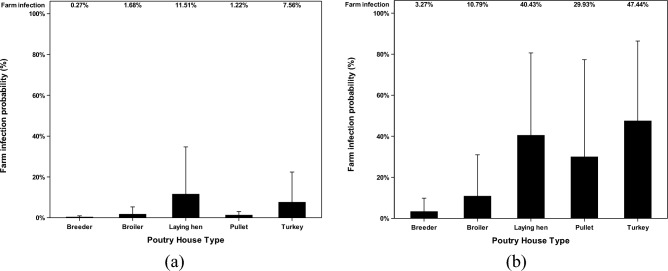
Farm infection probabilities of combined concentration of highly pathogenic avian influenza (HPAI) viruses at the recipient farms at different poultry house types. Farm infection probabilities of (**a**) combined AI concentration in default scenario, %; and farm infection probabilities of (**b**) combined AI concentration in ceiling scenario, %. The error bar stands for standard errors.

**Figure 9:**
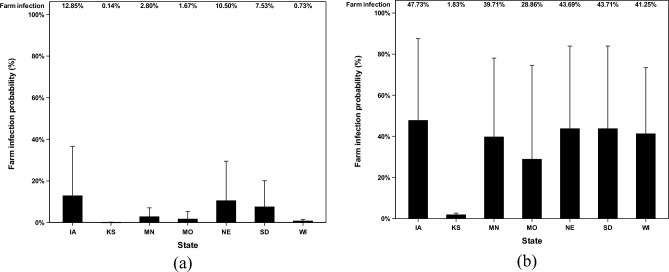
Farm infection probabilities of combined concentration of highly pathogenic avian influenza (HPAI) viruses at the recipient farms in different states. The farm infection probabilities of avian influenza (AI) viruses are reported according to two categories (default and ceiling). Farm infection probabilities of (**a**) combined AI concentration in default scenario, %; and farm infection probabilities of (**b**) combined AI concentration in ceiling scenario, %. The error bar stands for standard errors. X-axel abbreviations stand for states as follows IA (Iowa), KS (Kansas), MN (Minnesota), MO (Missouri), NE (Nebraska), SD (South Dakota), and WI (Wisconsin).

The farm infection probabilities caused by combined AI concentrations were reported in this study. The combined AI concentration is the combined concentration of airborne and deposited AI. The farm infection probabilities were reported based on different poultry house types (Fig. 8) and different states (Fig. 9). Airborne AI, after traveling for a long distance, gets in poultry facilities and stays both air suspended and deposited. The combined AI concentration simulated a more accurate situation happening in poultry facilities. With the combined concentration of AI, the farm infection probabilities are still low in the default scenario with the highest farm infection probabilities in laying hen facilities (11.9%) and in IA state (12.9%). In the ceiling scenario, the highest farm infection probabilities are in turkey facilities (47.4%) and in Iowa state (47.8%).

## Discussion

In this study, the HYSPLIT model was used to simulate the long-distance airborne transmission of HPAI and to assess the risk of airborne and deposited AI carried by poultry-litter dust particles. The study reported the viral concentrations simulated by HYSPLIT modeling in AI-infected poultry houses. The resulting hypothetical virus carried by PM_2.5_ concentrations generated by the HYSPLIT model was relatively low in all types of poultry farms. The concentration of airborne, deposited, and even combined concentration of airborne and deposited AI in these poultry farms were below the MIDa and MIDd values. Most poultry farms are hard to spread AI to surrounding poultry farms and causing infection. Compared to previous studies^11^, a similar conclusion was reported that the AI concentrations received by poultry farms from infected farms were much lower than the minimal infective dose. When traveling through the air for a long distance, the AI virus can be greatly affected by outdoor conditions. This causes them to be inactivated. A previous study^12^ reported that the AI virus could live for more than 100 days at 4 °C but was inactivated after 24 h at 28 °C and 30 min at 56 °C. The outbreak of AI in 2022 happened in late spring and early summer when the temperature was typically above 25 °C which can be a possible explanation for the short survival of the AI virus. The study also examined the farm infection probabilities at different farm locations. In the default scenario, when the conditions were more similar to the actual conditions, farm infection probabilities were generally low with the highest farm infection probabilities accounting for 14.8% in deposited AI and 2.1% in airborne AI. In the ceiling scenario, a remarkable farm infection probability was reported in South Dakota, which accounted for 79.0%. Although our results revealed that AI concentrations carried by PM_2.5_ were much lower than MID, the farm infection data in the ceiling scenario showed a significantly high probability (79.0%) of farm infection. The ceiling scenario stands for the worst situation when all conditions are optimal for the infection of airborne AI. The chance of a ceiling scenario is low.

Farm infection probabilities at different poultry house types were calculated in this study. In most scenarios, the turkey farm type showed the highest chance of being infected by long-distance airborne transmission of AI, except in default deposited and default combined, where the turkey farm type has the second highest chance of being infected by AI. These results are consistent with the real statistical report of infected farms according to USDA APHIS^4^. From February 08th to May 22nd, in total of 168 AI-infected cases, over 125 infected cases are turkey farms compared to 43 cases are from other poultry house types. The fact that the majority of turkey farms are infected by airborne AI might be explained by the ventilation rate (VR) of poultry farms. The typical ventilation rate for turkey is 9.2 m^3^ h^−1^ bird^−1^, for broiler breeder is 7.8 m^3^ h^−1^ bird^−1^, for broiler is 3.9 m^3^ h^−1^ bird^−1^, for laying hen is 2.0 m^3^ h^−1^ bird^−1^, and for pullet is 1.0 m^3^ h^−1^ bird^−1^^13–15^. The VR of turkey is higher than the VRs of other poultry house types. With the same number of birds and similar ventilation time, the turkey facilities get more air than other poultry house-type facilities. Thus, it increases the chance turkey farms get airborne AI transmitted from infected farms. In addition, the structure of turkey house makes the birds easier to expose to airborne AI. Turkey house type has an open sidewall structure which let the birds expose more to outside air^16^, while the laying hen house structure is closed. With this open structure of the house, turkeys have more chance to receive airborne AI than laying hens. Besides, bird susceptibility to the virus is also a factor that can affect the infection probability. A minimal infective dose is defined as the minimum number of infectious pathogens that causes infection/illness in birds. The MIDs were calculated^11^. Generally, the MIDs of turkeys were much lower than those of laying hens and other types of birds which implies that turkeys are much more susceptible to viruses than the other bird species. This factor can increase the chance of turkeys getting infected compared to the others.

In general, the infection probability estimated using deposited AI concentration, compared to that estimated using airborne AI concentration, was higher for all types of poultry. Deposited AI, after long-distance travel, can be deposited on any surface in the poultry farm and be picked up directly by birds. Meanwhile, airborne AI can infect birds by being inhaled by birds. Even though airborne AI can be suspended in poultry houses for long periods of time, the survivability may be compromised by the dehydration effect caused by the ventilation system which reduced the survivability of microorganisms in poultry facilities^17^. On the other hand, deposited AI can stay longer in poultry facilities by depositing on poultry litter or bird feces. This environment can provide preferential culture conditions for deposited AI, thus, supporting the survival of deposited AI. These discussions are consistent with previous studies. In a study conducted by Baleshwari^7^, authors reported that at 24 °C, the viral concentration in feces would reduce only about 20% after 24 h. While other authors^18, 19^ reported that human or pig-adapted influenza A virus subtypes showed a significant decrease (tenfold) in their viability in the air in 24 h. With better survivability, deposited AI had more chance to cause infection in birds rather than airborne AI. In addition, birds are often kept in confined spaces with limited ventilation and are in close contact with contaminated surfaces, feed, and water. The virus can then spread easily from bird to bird through contact with contaminated surfaces, feed, or water. In contrast, after getting in the poultry house, airborne AI requires the infected birds to be in close proximity to one another and for the virus to remain airborne for a sufficient period of time.

In the study, the type of poultry house, flock size, and distance from infected farms to recipient farms were the factors, besides the concentration of virus, that play a crucial role in the probability of infection in poultry. Larger flocks were found to have a higher chance of infection, while smaller flocks were at low infection risk. For instance, turkey poultry facilities generally have the highest chance of becoming infected. Yet, in the default deposited scenario, even though fewer laying hen farms were affected by AI, the infection probabilities of these farms still showed a higher chance than those of turkeys. This could be explained by that in this scenario, most of the infected laying hen farms were located in Iowa and Nebraska and had large ranges of flock sizes. The smallest flock had 915,900 birds, while the largest had 5,011,700 birds^20^. These flock sizes were significantly larger than those in turkey facilities. The study also found that as the distance from a source farm increases, the concentration of the virus decreases, reducing the probability of airborne infection. For instance, in the deposited ceiling scenario, the South Dakota farm infection probabilities were significantly high (about 79.0%). The infected and recipient farms were close to each other and mainly located in South Dakota, which made the recipient farms highly susceptible to the AI virus from infected farms.

Improper carcass management during AI outbreaks could lead to the spread of infections from infected farms to susceptible farms, potential for future legal liability, and public controversy^21^. To restrict the spread of the AI, infected farms are depopulated (euthanized). The depopulation will generate a considerable number of carcasses, as well as dust and manure. All of these wastes, if not being properly managed, can lead to the spread of airborne AI to susceptible farms. Glanville et al.^22^ reported that improper composting can result in the formation of airborne particles, spreading pathogens. In addition, garbage management can be an important risk factor for the spread of AI. If the trash collection site were not properly secured, it can cause virus leakage. Especially commercial poultry operation may share a common trash collection site^23^, this structure can post the risk of airborne spread of AI from the trash collection site to susceptible farms.

Although utilizing the HYSPLIT model can address the risk of long-distance travel of AI, this is a meteorological model which mainly focuses on the effects of meteorological factors on the virus. Some other factors including the interaction between dust particles or between dust particles and viruses, wild birds, humans, vehicles, and so on, were excluded from the model. In addition, the minimum accuracy distance of the model was 12 km, thus any farms within 12 km were not well simulated in the model. The HYSPLIT model helps in understanding the possibility and risk of airborne transmission in different locations as well as different poultry house types, but confirmed airborne transmission cannot be concluded without genetic analysis. In addition, the study reported the AI levels found in the recipient farms. The model predicted airborne viral concentrations of less than 10^–3.5^ EID_50_ m^−3^ in the default scenario, both for airborne and deposited AI. However, in real-life poultry houses, there is no such air sampler that can accurately collect viable airborne and deposited AI. As a result, it is challenging to confirm the accuracy of the meteorology model, including HYSPLIT. As a result, utilizing HYSPLIT may be a more sensitive method to explain and predict the potential outbreak of AI caused by airborne transmission. Finally, the findings of the infection probabilities in various poultry house types and across different states can provide a comprehensive understanding of which poultry house types have a high chance of being exposed to airborne avian influenza, thereby allowing for proper preventive measures to be taken.

## Methods

### Infected farm data

Data from 168 infected cases in the U.S. were obtained from the Animal and Plant Health Inspection Service (APHIS) and Watt Poultry (WattPoultry.com). Each confirmed case datum included physical address, county and state, infection confirmation date, and the number of birds infected. In this study, 168 infected commercial poultry cases (72% of the national total of commercial farm infections) in the Mid-Western U.S. were focused on, and data from the period of February 08th, 2022 to May 22nd, 2022, were included in the model. The dispersion simulation modeling was performed to examine if the confirmed cases in the Mid-West received air from other farms before being infected and estimate the concentration of airborne AI received. The reason for selecting data from the commercial farms and excluding data from backyark farms in the Mid-Western area was that although the number of backyard flocks infected was higher than commercial flocks, the number of infected birds per backyard flock was significantly lower than the number of birds infected per commercial farm^20^.

### HYSPLIT modeling

The HYSPLIT model (Hybrid Single-Particle Lagrangian Integrated Trajectory model, National Oceanic and Atmospheric Administration, Washington, D.C., U.S) is a computational model that is utilized to calculate the trajectory of air parcels. This helps to establish both the direction and distance that a particular air parcel, along with any associated air pollutants, will travel. HYSPLIT modeling can also estimate air pollutant dispersion, chemical transformation, and deposition. In this study, the HYSPLIT model was used to simulate the air movement of PM_2.5_ which hypothetically carries AI to examine if the PM_2.5_ particles travel passing through other farms before the farms became infected. The modeling also computed the concentration of airborne and deposited AI carried by PM_2.5_ dust particles (or fine dust particles) in the farms. The AI survival time of 24 h at 28 °C and a period of 21 days prior to the infection confirmation dates^11^ was applied in the modeling. Three different periods, namely 8 a.m., 4 p.m., and 12 a.m. (Local Standard Time) were calculated separately to reduce the temporal wind speed and direction-varied effects. Airborne and deposited AI were examined at the height of 6 m above ground level (m agl), considering the typical height of the poultry houses. The height of typical air inlets which is 1.5 m was applied in the model.

The concentration of airborne AI carried by PM_2.5_ was assessed by using both default and ceiling input data in the HYSPLIT model. The AI concentration has been reported to be detected predominantly from fine dust particles^8^, and with the long range of transmission, the PM_2.5_ size can be a big concern for public health. The default data stands for the representative data from scientific references, and the ceiling data stands for parameters that might happen in the worst scenarios. The required parameters were provided in Table S1.

### Model processing

Forward concentration modeling was used to assess the possibility of infections from infected poultry farms to other farms. First, the AI data collected daily from APHIS and Watt Poultry websites were imported into HYSPLIT modeling. The AI data were divided into four categories including default PM_2.5_, ceiling PM_2.5_, deposited default PM_2.5_, and deposited ceiling PM_2.5_. Default PM_2.5_ and ceiling PM_2.5_ stand for the airborne AI concentration carried by PM_2.5_ in the default scenario and worst scenario, respectively. Deposited default PM_2.5_ and deposited ceiling PM_2.5_ are the AI concentration carried by PM_2.5_, after being aerosolized, being deposited on the surface of poultry facilities in the default scenario and worst scenario respectively. Hypothetically, the deposited AI, after being transmitted into the poultry house and deposited on surfaces, would be picked up by birds in the poultry house. Meteorological data was downloaded from the National Oceanic and Atmospheric Administration (NOAA, Washington, D.C., U.S.) website for each day. To reduce the variability of wind direction as well as meteorological conditions, the model is run every 8-h interval which results in three trajectories covering the air arrival time at 8 am, 4 pm, and 12 am Local Standard Time (LST). After processing the data, airborne AI concentration data collected from the modeling were then exported as kmz files which were then loaded in Google Earth Pro (Google LLC, Mountain View, CA, U.S.). With concentration modeling, the clear viral pattern movement of AI can be observed on Google Earth (video V1). The AI concentration data corresponding to the 4 scenarios and types of poultry farms would be reported. The modeling concentrations of AI were then compared to the minimal infective doses of airborne AI viruses and deposited AI viruses calculated based on formulas (1) (2) and were utilized to assess the possibility of infection for each case.

### Minimal infective doses for airborne transmission

Minimal infective dose for airborne transmission (MIDa) is the quantity of airborne AI (measured in EID_50_ m^−3^) that is necessary to cause infection in a healthy bird. The 50% egg infective dose (EID_50_) or egg/embryo infective dose 50 is a unit for the concentration of a certain virus. Specific pathogen-free (SPF) eggs/embryos are employed as the culture medium. The original viral sample is serially diluted first. Each dilution is injected into a small number of eggs. Then, this dilution is used to determine the EID_50_ when 50% of the eggs in a dilution are infected. The MIDa were calculated based on the general minimal infective dose (MIDt). MIDt were 10^3^ EID_50_ for turkeys^24^ and 10^3.5^ EID_50_ for laying hens^25^. The same MIDa was used for laying hens, broilers, breeders, and pullets. The MIDa values were calculated by the following formula (1):

1 $$\mathrm{MIDa }=\mathrm{ MIDt}\times \frac{1}{v \times r \times 24 },$$

where MIDt is the general infective dose, EID_50_; v is the tidal volume of the bird, m^3^; r is respiratory rate, time h^−1^; and MIDa is the Minimal infective dose for airborne transmission, EID_50_ m^−3^ for a day (24 h).

Minimal infective dose for deposited (MIDd) AI is the quantity of deposited AI (measured in EID_50_ m^−2^) that is necessary to cause infection in a healthy bird. The same MIDd was used for laying hens, broilers, breeders, and pullets. The MIDd values were calculated by the following formula (2):

2 $$\mathrm{MIDd }=\mathrm{ MIDt}\times \frac{1}{s},$$

where MIDt is the general infective dose, EID_50_; s is the area that a bird needs in the house, 0.11 m^2^ per bird (or stocking density of 9 birds per m^2^) for laying hen^26^ and 0.35 m^2^ per bird (or stocking density of about 3 birds per m^2^) for turkey^27^, m^2^ bird^−1^; and MIDd is the Minimal infective dose for deposited AI viruses, EID_50_ m^−2^.

### Farm infection probability

The probability of farm infection (formula 4) is determined by the probability of individual-bird infection (formula 3) at a given dosage (d) and flock size (nf). We assumed that 95% of birds might get infected at the (d) dosage, θ was 0.00069 for turkeys and 0.00022 for laying hens^11^.

3 $$Pi = { 1 } - \, ({1 } - \theta )^{{\text{d}}} ,$$

4 $$Ph = { 1 } - \, \left( {{1 } - Pi} \right)^{{{\text{nf}}}} ,$$

where Pi is probability of individually bird infection, %; θ is probability of one ID_50_ infect to bird, %; d is dosage of viruses exposed to a bird, EID_50_ bird^−1^ day^−1^; Ph is is probability of farm infection, %; nf is size of flock, number of birds.

### Supplementary Information

Supplementary Information.

Supplementary Video 1.

## Data Availability

The data of this study are available on the Animal and Plant Health Inspection Service (APHIS) and Watt Poultry websites.
