# Supplementary material for: Modeling long-distance airborne transmission of highly pathogenic avian influenza carried by dust particles
Source: Sci Rep. 2023 Sep 27;13:16255. doi: 10.1038/s41598-023-42897-2 (PMC10533824; doi:10.1038/s41598-023-42897-2)
Supplement: Supplementary file 1 — Supplementary Information. [file 41598_2023_42897_MOESM1_ESM.docx]

**Supporting information**

**Modeling Long-Distance Airborne Transmission of Highly Pathogenic Avian Influenza Carried by Dust Particles**

# X. D. Nguyen^1^, Y. Zhao^1,*^, J. Lin^1,+^, J. L. Purswell^2,+^, T. Tabler^1,+^, B. Voy^1,+^, S. Hawkins^3,+^, and J. D. Evans^2,+^

*^1^Animal Science, The University of Tennessee, Knoxville, USA; ^2^Poultry Research Unit, USDA Agricultural Research Service, Mississippi State, USA; ^3^Biosystems Engineering and Soil Sciences, The University of Tennessee, Knoxville, USA; ^*^* *Corresponding author: Yang Zhao,* [*yzhao@utk.edu*](mailto:yzhao@utk.edu)*; ^+^these authors contributed equally to this work*

**Table S1. Input parameters used for concentration modeling in the HYSPLIT model.**

| Parameter | Unit | Value |
| --- | --- | --- |
| Total run time per cycle | h | 24 |
| Trajectory modeling direction | - | Forward |
| Top of the model | m | 1,500 |
| Incubation period | day | 7 [1] |
| Virus emission duration | h | 24 |
| Height of concern | m | 0-6 (airborne) & 0 (deposit) |
| PM_2.5_ emission rate | mg/bird/d | 1 (laying hen) [2-4] |
|  |  | 38 (turkey) [2-4] |
| PM density | g/cm^3^ | 1.5 [5] |
| PM deposition velocity | m/s | 0.001 (PM_2.5_) [6] |
| Half-life | day | 1.0/1.5 (default/ceiling) [7] |
| Percentage of manure in dust | % | 5 (laying hen) [4,8]  40 (turkey) [4,8] |
| Viral shedding rate | Log EID_50_/[g feces] | 4/5 (default/ceiling) [9] |
| Viral survival reduction | % | 60 (after 24 h) [10] |

**Table S2. Summary of 168 infected cases during 2015 HPAI outbreak. Data from 168 infected cases were collected from the Animal and Plant Health Inspection Service (APHIS) and Watt Poultry (WattPoultry.com). Data from February 08^th^, 2022 to May 22^nd^, 2022 were utilized.**

| Parameter | Value |
| --- | --- |
| **Total cases/farms** | 168 |
| **Type** | Turkey – 125 cases  Layer – 14 cases  Pullet – 6 cases  Broiler – 8 cases  Breeder – 15 cases |
| **First date** | 2/8/2022 (Turkey) |
| **Last date** | 5/21/2015 (Turkey) |
| **Location** | Mid-Western U.S. |
| **States included** | Iowa, Kansas, Minnesota, Missouri, Nebraska, South Dakota, Wisconsin |

**Video V1. Concentration modeling for airborne virus carried by PM_2.5_ during 2022 HPAI outbreak. The clear viral pattern movement of AI can be observed on Google Earth. Default input values are used for concentration modeling. The light blue area shows the virus concentration in a range of 10^-9^ 50% egg infective dose per cubic meter (EID_50_m^-3^). The green area shows the virus concentration in a range of 10^--8^ 50% egg infective dose per cubic meter (EID_50_m^-3^). The dark blue area shows the virus concentration in a range of 10^-7^ 50% egg infective dose per cubic meter (EID_50_m^-3^). The yellow area shows the virus concentration in a range of 10^-6^ 50% egg infective dose per cubic meter (EID_50_m^-3^). The highlighted locations on Google map were HPAI confirmed cases.**

**References**

1. USDA, A.a.P.H.I.S.A.-. *Case Definition - Avian Influenza (AI)*; Animal and Plant Health Inspection Service - USDA: <https://www.aphis.usda.gov/animal_health/monitoring_surveillance/avian-influenza-case-definition.pdf>, 2022.

2. Shepherd, T.A., Zhao, Y., Li, H., Stinn, J.P., Hayes, M.D. & Xin, H. Environmental assessment of three egg production systems—Part II. Ammonia, greenhouse gas, and particulate matter emissions. *Poultry science* **2015**, *94*, 534-543, doi:<https://doi.org/10.3382/ps/peu075>.

3. Li, H., Xin, H., Burns, R.T., Jacobson, L.D., Noll, S., Hoff, S.J., Harmon, J.D., Koziel, J.A. & Hetchler, B.P. Air emissions from tom and hen turkey houses in the US Midwest. *Transactions of the ASABE* **2011**, *54*, 305-314, doi:10.13031/2013.36258.

4. Cambra-López, M., Aarnink, A.J.A., Zhao, Y., Calvet, S. & Torres, A.G. Airborne particulate matter from livestock production systems: A review of an air pollution problem. *Environmental pollution* **2010**, *158*, 1-17, doi:<https://doi.org/10.1016/j.envpol.2009.07.011>.

5. Rosenthal, E., Schneider, T., Büscher, W. & Diekmann, B. Sedimentation von tierartspezifischen Stäuben im Stallinnenraum. *Landtechnik* **2007**, *62*, 102-103.

6. Lin, J.J., Noll, K.E. & Holsen, T.M. Dry deposition velocities as a function of particle size in the ambient atmosphere. *Aerosol Science and Technology* **1994**, *20*, 239-252.

7. Shaman, J. & Kohn, M. Absolute humidity modulates influenza survival, transmission, and seasonality. *Proceedings of the National Academy of Sciences* **2009**, *106*, 3243-3248.

8. Zhao, Y., Aarnink, A., De Jong, M. & Groot Koerkamp, P.W.G. Airborne Microorganisms From Livestock Production Systems and Their Relation to Dust. *Critical Reviews in Environmental Science and Technology* **2014**, *44*, doi:<https://doi.org/10.1080/10643389.2012.746064>.

9. Forrest, H.L., Kim, J.-K. & Webster, R.G. Virus shedding and potential for interspecies waterborne transmission of highly pathogenic H5N1 influenza virus in sparrows and chickens. *Journal of virology* **2010**, *84*, 3718-3720.

10. Kurmi, B., Murugkar, H.V., Nagarajan, S., Tosh, C., Dubey, S.C. & Kumar, M. Survivability of Highly Pathogenic Avian Influenza H5N1 Virus in Poultry Faeces at Different Temperatures. *Indian journal of virology : an official organ of Indian Virological Society* **2013**, *24*, 272-277, doi:10.1007/s13337-013-0135-2.
